# Prevalence and perinatal outcomes of non-communicable diseases in pregnancy in a regional hospital in Haiti: A prospective cohort study

**DOI:** 10.7189/jogh.11.04020

**Published:** 2021-04-17

**Authors:** Isabelle Malhamé, Rodney Destiné, Widmise Jacquecilien, Bidjinie H Coriolan, Wacquinn St-Loth, Marie Claudy Excellent, Benjaminel Scaide, Remy Wong, Sarah Meltzer, Eddy Jean-Baptiste, Louise Pilote, Julia E von Oettingen, Kerling Israel

**Affiliations:** 1 Department of Medicine, McGill University Health Centre, Montreal, Canada; 2 Department of Medicine, Saint-Nicolas Hospital, Saint-Marc, Haiti; 3 Department of Obstetrics and Gynecology, Saint-Nicolas Hospital, Saint-Marc, Haiti; 4 Department of Paediatrics, Saint-Nicolas Hospital, Saint-Marc, Haiti; 5 Fondation Haïtienne de Diabète et de Maladies Cardiovasculaires, Port-au-Prince, Haiti; 6 Department of Paediatrics, McGill University Health Centre, Montreal, Canada

## Abstract

**Background:**

The prevalence of non-communicable diseases (NCDs) is rising in low and middle-income countries (LMIC). We aimed to report on the prevalence of NCDs in pregnancy and their associated perinatal outcomes in a regional hospital in Haiti.

**Methods:**

We conducted the “Diabète et hYpertension Artéerielle et leurs issues MAternelles et Néonatales” (DYAMAN) prospective cohort study in a regional hospital in Haiti. Pregnant women presenting to care at 24-28 weeks were screened and treated for diabetes (DM) and hypertensive disorders of pregnancy (HDP) using setting-adapted protocols. Prevalence of NCDs and associated maternal-neonatal outcomes were described.

**Results:**

715 women were included, of which 51 (7.1%) had DM, 90 (12.6%) had HDP, and 30 (4.2%) had both DM and HDP (DM/HDP). Of 422 (59%) women delivered in hospital, 58 (13.7%) had preeclampsia, including 5 (8.6%) with eclampsia. Preterm birth <32 weeks was more common in the HDP than the control, DM, and DM/HDP groups. More low birth weight babies (n = 20, 25.6%) were born to the HDP group than to the control (n = 20, 7.1%), DM (n = 1, 2.7%), and DM/HDP (n = 3, 12%) groups (*P* < 0.001). Macrosomia and hypoglycemia affected 5 (8%) neonates of women with DM. Perinatal mortality, affecting 36/1000 births, was mainly driven by maternal NCDs.

**Conclusions:**

NCDs in pregnancy led to adverse maternal and perinatal outcomes. This study will help to prepare future refinements aimed at optimizing the management of NCDs in pregnancy in LMIC. Research is required to understand barriers to patient attendance at antenatal follow-up, treatment escalation for hyperglycemia, and in-hospital delivery.

Non-communicable diseases (NCDs) account for 63% of the worldwide mortality [1]. Importantly, 80% of these deaths occur in low and middle-income countries (LMIC) [1,2]. Evidence-based interventions to prevent and manage NCDs in LMIC are urgently needed to face their projected rising incidence [1]. Yet, research on the epidemiology, determinants, care delivery aspects, and outcomes of NCDs in low-resource settings is disproportionately scarce. NCDs in pregnancy include pre-existing and gestational diabetes mellitus (DM) as well as hypertensive disorders of pregnancy (HDP). These NCDs are associated with adverse maternal, pregnancy, and perinatal outcomes [3,4] and carry long-term risks of cardiovascular morbidity for both mothers and their offspring [5-7]. As a result, developing and evaluating setting-adapted screening and management programs for NCDs in pregnancy are among the World Health Organization’s (WHO) research priorities [8].

The maternal mortality ratio in Haiti is estimated at 359 per 100 000 live births, representing the highest ratio in the Western hemisphere [9]. Neonatal mortality at 28 per 1000 live births remains one of the highest in the world [10]. Despite this high maternal-neonatal mortality, information on national prevalence of NCDs in pregnancy and their impact on maternal and neonatal outcomes is lacking. Indeed, while most women in Haiti are screened at least once for HDP during their antepartum follow-up [11], a national screening and treatment program for gestational DM has not yet been implemented.

We conducted the “Diabete et hYpertension Arterielle et leurs issues MAternelles et Neonatales” (DYAMAN) study, a collaborative research initiative between Haitian and Canadian health care providers and investigators aiming to contribute further evidence on the prevalence of NCDs in pregnancy and their associated perinatal outcomes in a regional hospital in Haiti.

## METHODS

### Ethics considerations

Written informed consent for participation was obtained after explaining aims, format, and risks of the study to eligible women in either Haitian Creole or French language, according to patient preference. For patients not able to read, the consent form was read aloud. Patients unable to write signed by marking a cross in the presence of a witness who co-signed the consent form. A study coordinator fluent in both languages was available for questions during the consent process.

### Study population

We conducted a prospective cohort study at the Saint-Nicolas Hospital, in the community of Saint-Marc, in the Artibonite region of the republic of Haiti between June 1, 2017 and February 28, 2019. Women presenting to routine care at the hospital’s antenatal clinic between 24 and 28 weeks of gestation were eligible. Potential participants were informed about the study during their scheduled appointment with a midwife and were then approached by study personnel. Women with a pregnancy of less than 24 weeks of gestational age and women who were not fasting yet interested in participating in the study were given the opportunity to enroll later. Women <18 years of age and those who were not pregnant were excluded.

At time of cohort entry, research personnel fluent in French and Haitian Creole administered an initial survey. This survey was comprised of demographic information (including age, education, profession, marital status, neighborhood of residence, living situation), past obstetrical history (including date of last menstruation period, current gestational age, number of prior pregnancies and prior abortions, prior history of fetal demise, preterm delivery, small for gestational age baby, or hypertensive disorders of pregnancy and gestational diabetes), past medical history (including known hypertensive disorder, type 1 or type 2 diabetes, history of cerebrovascular accident, history of anemia, and known HIV status), family history (including maternal history of hypertensive disorder of pregnancy, diabetes, or cardiac history), and social history (including number of daily meals, and history of tobacco smoking and alcohol consumption). Anthropometric measurements were also recorded (including weight, height, and fundal height measured by obstetric midwives with metric-based measurement tape); body mass index (BMI) was calculated.

### Screening for gestational diabetes and hypertensive disorders of pregnancy

A one-step oral glucose tolerance test (oGTT) for the diagnosis of gestational diabetes mellitus was performed using a 75 g oral glucose solution (Glucose-75, JAMP, Boucherville, Canada) [12]. This consisted in 300 mL of oral beverage. We measured glycemia at time 0, 1h, and 2h after drinking the oral glucose solution with a high-precision, high-accuracy portable glucometer (Countour Next, Ascensia Diabetes Care, NJ, USA) [13,14]. We opted for glucometer-based capillary glucose measurements as laboratory-based serum glucose analysis is seldom available at the regional hospital (as is the case for most public health care facilities in Haiti) [14,15]; an approach that has previously been shown to be accurate in settings were laboratory glucose measurements are not reliably available [16]. Women with fasting glycemia >92 mg/dL (5.1 mmol/L), 1h glycemia >180 mg/dL (10 mmol/L), or 2h glycemia >153 mg/dL (8.5 mmol/L) were considered as having gestational DM [12]. Women with preexisting diabetes or women with fasting glycemia >180 mg/dL did not undergo further oGTT and were considered as having pre-existing DM.

Blood pressure (BP) measurements were taken twice at rest, 15 minutes apart, and averaged using an electronic sphygmomanometer (OMRON M7, OMRON, Kyoto, Japan) validated for use in pregnancy [17]. Urinary dipstick testing assessed proteinuria. Hypertensive disorders included chronic hypertension, gestational hypertension, and preeclampsia. Women were considered as having chronic hypertension if they reported a diagnosis of hypertension or were treated for hypertension <20 weeks of gestation. They were considered as having gestational hypertension if they had persistent systolic BP≥140 mm Hg or a diastolic BP≥90 mm Hg at ≥20 weeks of gestation [18]. Preeclampsia was diagnosed when chronic or gestational hypertension was accompanied by new onset proteinuria, pulmonary edema, liver enzyme abnormalities, thrombocytopenia, neurologic symptoms, intractable hypertension, or fetal distress [4,18]. Women without DM and without HDP were considered as the control group.

### Antepartum management

Follow-up visit frequency was determined by study group (Appendix S1 in the **Online Supplementary Document**). Women could change groups during follow-up if they had a new NCD diagnosis.

Clinical protocols for the management of DM were co-developed by Haitian and Canadian investigators adapted from international society guidelines from the International Federation of Gynecology and Obstetrics (FIGO), the Canadian Diabetes Association (CDA), and the National Institute for Health and Care Excellence (NICE) [5,15,19]. Women with DM received nutrition and exercise counseling by a specialized diabetes nurse. They were given portable glucometers (Contour Next, Ascensia Diabetes Care, NJ, USA) for home glucose monitoring. Glycemic targets were 80-90 mg/dL (4.4-5.0 mmol/L) fasting and 110-140 mg/dL (6.1-7.8 mmol/L) 1 hour after a meal. Participants with fasting glycemia <92 mg/dL (5.1 mmol/L) were treated with non-pharmacologic measures only; those with fasting glycemia 92-126 mg/dL (5.1 and 7.0 mmol/L) were also treated with metformin, with a targeted dose of 1g twice daily; those with fasting glycemia >126 mg/dL (7.0 mmol/L) required both metformin and nightly subcutaneous insulin NPH (Appendix S2 in the **Online Supplementary Document**). If the postprandial glycemia was >150 mg/dL twice in a week, insulin R was given with meals. Women were followed at least weekly initially (Appendix S1 in the **Online Supplementary Document**).

As per local protocols already in place at the beginning of the study conduct, women with BP≥160/110 mm Hg were admitted for inpatient monitoring and management of severe hypertension. Systolic BP targets were 130-155 mm Hg and diastolic BP targets were 80-105 mm Hg. Medications locally available for use included intravenous labetalol and hydralazine, short acting nifedipine, as well as oral methyldopa, labetalol, and long acting nifedipine.

### Peripartum management

Women with DM were managed according to a local intrapartum protocol using subcutaneous insulin R every 2h (Appendix S3 in the **Online Supplementary Document**). Women with HDP had frequent BP monitoring, and were given intravenous or intramuscular magnesium sulfate, if indicated (Figure S4 in the **Online Supplementary Document**). Information was collected on delivery mode, obstetrical complications, glycemia, BP measurements, neonatal birth weight, and neonatal complications. Fetal and maternal deaths were also recorded.

### Neonatal management

The pediatric team assessed infants of mothers with DM, and those of mothers with HDP if they were born with low birth weight (birth weight <2500 g), macrosomia (birth weight >4000 g), or if they were born <37 weeks of gestation. Small for gestational age (SGA) was defined as birth weight lower than the 10th percentile for gestational age. First line therapy for neonatal hypoglycemia was maternal breastfeeding or formula milk followed by either continued oral nutrition or intravenous dextrose according to severity of hypoglycemia (Appendix S4 in the **Online Supplementary Document**). Neonatal deaths were recorded.

### Data collection

Outpatient data was prospectively collected by the study nurse at every outpatient study visit. Inpatient data was retrospectively collected from a detailed medical chart review. Study data were collected and managed using REDCap electronic data capture tools hosted at the Research Institute of the McGill University Health Centre [20,21].

### Statistical analysis

Clinical characteristics were described using standard descriptive measures. Analyses were performed across predefined diagnosis groups (ie, control, DM, HDP, DM/HDP groups). Comparisons between continuous and categorical variables were made with analysis of variance (ANOVA) and Fisher’s exact test, respectively. Assuming prevalence estimates of gestational diabetes between 10 and 15%, at a precision of 5%. and with a 95% confidence interval, we estimated that a sample size of 657 women was required given an oGTT’s sensitivity and specificity of 0.7 and 0.9. Oversampling of 5%-10% was foreseen to account for loss to follow-up. A *P* value ≤0.05 was considered statistically significant. All statistical analyses were performed with RStudio (version 1.1.383, Boston, MA, USA).

## RESULTS

### Study population and baseline characteristics

**Figure 1:**
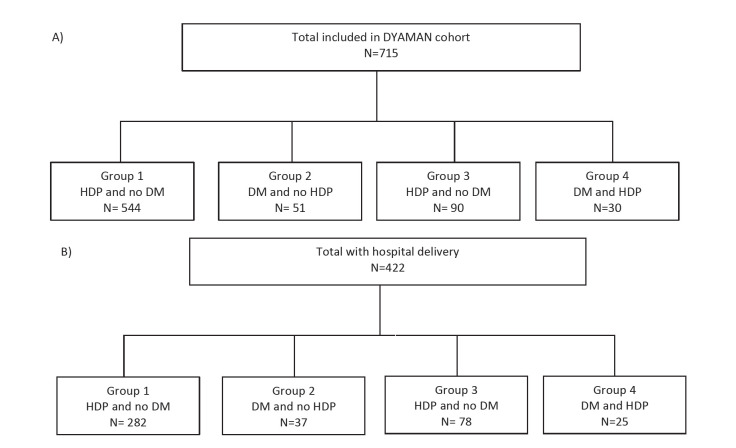
Flowchart of the study population. **Panel A.** Flow chart for the DYAMAN cohort. **Panel B.** Flow chart for women of the DYAMAN cohort who delivered in hospital.

In total, 715 women aged 29 years on average, were included in the DYAMAN cohort, comprising 544 (76.1%) controls, 51 (7.1%) women with DM only, 90 (12.6%) women with HDP only, and 30 (4.2%) women with DM/HDP (**Table 1** and **Figure 1**, Panel A). In total, DM affected 81 (11.3%) women and HDP affected 120 (16.8%) women. Women with DM were more likely to be of maternal age ≥35 years than controls, and women with HDP (**Table 1**). Women with HDP had higher rates of obesity than controls, whereas women with DM did not (**Table 1**). Alcohol use was more common among women with than without DM. Moreover, women with DM were more likely to have 0 years of education than women without DM. Although most women lived in houses built with concrete, at least a quarter of them were affected by food insecurity (**Table 1**). Missing values for variable included in Table 2 can be found on Table S1 in the **Online Supplementary Document**.

**Table 1:** Descriptive characteristics of the study population

Total, n = 715	Control, (N = 544)	DM, (N = 51)	HDP, (N = 90)	DM/HDP, (N = 30)	*P-*value
Gestational age at initial visit, weeks, mean ± SD	26.5 ± 1.6	26.3 ± 1.8	26.7 ± 1.5	26.1 ± 1.5	0.405
Age, mean ± SD	28.2 ± 6.1	30.8 ± 7.5	29.8 ± 5.5	32.1 ± 6.2	<0.0001
Maternal age ≥35 years, n (%)	79 (14.5)	14 (27.5)	14 (15.9)	10 (33.3)	0.011
BMI, mean ± SD	26.3 ± 4.9	26.9 ± 5.2	27.7 ± 5.8	29.4 ± 6.3	0.002
-Underweight (BMI <18.5), n (%)	6 (1.1)	2 (3.9)	1 (1.1)	1 (3.3)	0.164
-Obesity (BMI ≥30), n (%)	109 (20)	8 (15.7)	31 (34.4)	13 (43.3)	<0.001
Prior pregnancies, number, mean ± SD	1.3 ± 1.4	1.5 ± 1.6	1.4 ± 1.4	2.2 ± 2.0	0.008
Live children, number, mean ± SD	1.6 ± 1.2	1.6 ± 1.6	1.5 ± 1.1	1.9 ± 1.9	0.646
Matrimonial status:					
Married, n (%)	196 (36)	23 (45.1)	35 (38.9)	10 (33.3)	0.580
Living with partner, n (%)	174 (32)	16 (31.4)	32 (35.6)	14 (46.7)	0.376
Single, n (%)	173 (32)	12 (23.5)	23 (25.6)	6 (20)	0.269
Education length, year, mean ± SD	11.0 ± 3.7	10.4 ± 4.5	11.1 ± 3.6	9.7 ± 4.5	
0 years of education, n (%)	11 (2)	4 (7.8)	2 (2.2)	2 (6.7)	0.036
<10 years of education, n (%)	142 (26.1)	11 (21.6)	21 (23.3)	11 (36.7)	0.480
House built with concrete, n (%)	475 (87.3)	45 (88.2)	80 (88.9)	29 (96.7)	0.063
Food insecurity, n (%)	147 (27)	14 (27.5)	23 (25.6)	9 (30)	0.960
Current smoker, n (%)	3 (0.6)	0	0	0	1
Current alcohol use, n (%)	19 (3.5)	5 (9.8)	6 (6.7)	1 (3.3)	0.099
Comorbidities:					
One elevated blood pressure in lifetime, n (%)	32 (5.9)	1 (2.0)	21 (23.3)	11 (36.7)	<0.001
-Preexisting diabetes, n (%)	1 (0.2)	7 (13.7)	0	5 (16.7)	<0.001
Obstetrical history:*					
Gestational hypertension, n (%)	37 (11.8)	3 (10.0)	23 (40.4)	6 (30.0)	<0.001
Preeclampsia, n (%)	12 (3.8)	1 (3.3)	11 (19.3)	2 (10.0)	0.001
Prior testing for gestational diabetes, n (%)	32 (10.2)	3 (10.0)	6 (10.5)	2 (10.0)	1.000

BMI – body mass index, DM – diabetes Mellitus, HDP – hypertensive disorder of pregnancy, SD – standard deviation

*Obstetrical history was obtained from women who had delivered at least once.

**Table 2:** Clinical parameters during antenatal follow-up

Attendance	Initial visit (n = 715)	Visit 2 (n = 604)	Visit 3 (n = 489)	Visit 4 (n = 313)	Visit 5 (n = 144)	Visit 6 (n = 63)	Delivery (n = 422)
Gestational age, week, median (IQR)	26.7 (25.1-28.0)	31 (29.6-32.6)	34.7 (32.9-36.1)	36.9 (35.0-38.3)	38 (36-39.4)	38.4 (35.3-40)	39 (37.8-40.2)
Control, n (%)							
-DM, n (%)	71 (9.9)	65 (10.8)	58 (11.9)	53 (16.9)	44 (30.6)	33 (52.4)	42 (10.0)
-HDP, n (%)	29 (4.1)	21 (3.5)	19 (3.9)	22 (7.0)	9 (6.3)	3 (4.8)	60 (14.2)
-DM/HDP, n (%)	10 (1.4)	10 (1.6)	10 (2.0)	9 (2.9)	6 (4.2)	5 (7.9)	20 (4.7)
Total patients with DM (N = n present/n expected)	N = 81/81	N = 75/81	N = 68/81	N = 62/81	N = 50/81	N = 38/81	N = 62/81
-Minimum fasting BG, mg/dL, median (IQR)	–	74(66-84)	76 (70-83)	73 (69-81)	76 (71-81)	73 (68-79)	–
-Maximum fasting BG, mg/dL, median (IQR)	–	110 (93-135)	106 (95-131)	105 (93-128)	97 (88 -117)	102 (90-116)	–
-Minimum after 1h, mg/dL, median (IQR)	–	91 (78-101)	85 (73-99)	84 (74-95)	84 (77-99)	90 (77-96)	–
-Maximum after 1h, mg/dL, median (IQR)	–	150 (127-173)	142 (126-182)	150 (128-179)	154 (124-172)	150 (124-184)	–
-Diet only, n (%)*	N/A	43 (57.3)	42 (61.8)	37 (59.7)	29 (58.0)	20 (52.6)	–
-Metformin, n (%)*	11 (13.6)	21 (28.0)	20 (29.4)	14 (22.6)	14 (28.0)	12 (31.6)	–
-Insulin, n (%)*	1 (1.2)	1 (1.3)	1 (1.5)	1 (1.6)	1 (2.0)	0	–
-Metformin and insulin, n (%)*	0	2 (2.7)	3 (4.4)	6 (9.7)	4 (8.0)	4 (10.5)	–
-Metformin changed to insulin, n -(%)*	N/A	2 (2.7)	1 (1.5)	3 (4.8)	0	0	–
-Daily dose of metformin (mg/d)	473 ± 75	413 ± 122	448 ± 144	453 ± 98	486 ± 160	469 ± 125	–
-Daily dose of insulin (Units/d)	8 ± 4	6 ± 1	8 ± 3	8 ± 3	8 ± 3	6 ± 2	–
Total patients with HDP (N = n present/n expected)	N = 39/39	N = 31/50	N = 29/59	N = 31/68	N = 15/68	N = 8/69	N = 103/120
-Systolic blood pressure, mean ± SD	115 ± 17	115 ± 17	118 ± 16	121 ± 17	120 ± 15	118 ± 17	143.4 ± 19.2
-Diastolic blood pressure, mean ± SD	72 ± 13	72 ± 13	75 ± 13	78 ± 13	79 ± 13	77 ± 11	90.0 ± 15.8
-Monotherapy, n (%):†	27 (84.4)	18 (58.1)	20 (69.0)	16 (51.6)	10 (66.7)	4 (50.0)	–
Methyldopa, n (%)†	18 (56.3)	15 (48.3)	16 (55.2)	15 (48.4)	10 (66.7)	4 (50.0)	–
Nifedipine, n (%)†	0	0	0	0	0	0	–
Other, n (%)†	8 (25.0)	3 (9.7)	4 (13.8)	1 (3.2)	0	0	–
-Dual therapy, n (%)†	1 (3.1)	0	0	0	0	0	–
Preeclampsia, n (%)	–	–	–	–	–	–	58 (13.7)

*Denominator composed of women with DM and DM/HDP present at clinic visit.

†Denominator composed of women with HDP and DM/HDP.

**Figure 2:**
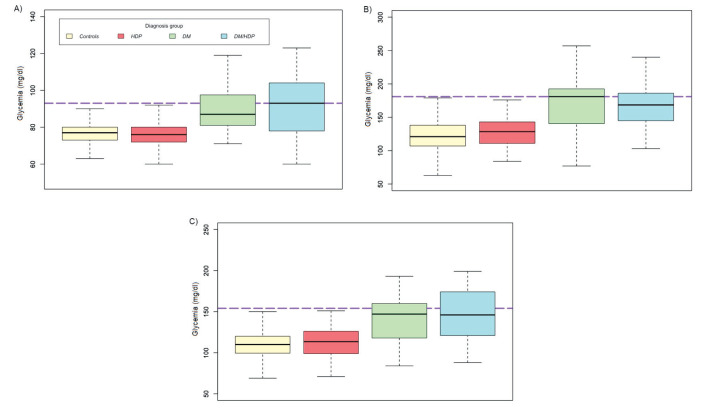
Glycemia at 0, 1, and 2 hours during oral glucose tolerance test. **Panel A.** Fasting glycemia during oral glucose tolerance test. **Panel B.** Glycemia at 1 hour during oral glucose tolerance test. **Panel C.** Glycemia at 2 hours during oral glucose tolerance test.

At time of diagnostic testing for DM, women with DM/HDP tended to have higher fasting glycemia than women with DM alone (median DM = 87 mg/dl vs DM/HDP = 93 mg/dl, *P* < 0.41, **Figure 2**, Panel A), whereas women with DM tended to have higher postprandial glycemia at 1h (median DM = 181 mg/dl vs DM/HDP = 168.5 mg/dl, *P* < 0.824, **Figure 2**, Panel B). No differences were found among women with HDP and controls at 1h (median HDP = 128.5 mg/dl vs control = 121 mg/dl, *P* = 0.056) and 2h (median HDP = 113.5 mg/dl vs control = 110 mg/dl, *P* = 0.288, **Figure 2**, Panels B and C).

### Clinical parameters during antepartum follow-up

Attendance at the antepartum clinic ranged from 63 (8.8%) to 604 (84.5%) patients per visit (**Table 2**). On average, women in the control group had 4.7 ± 1.8 of 6 foreseen visits, and women in the DM, HDP, and DM/HDP groups had 8.0 ± 2.5, 5.2 ± 2.1, and 8.2 ± 2.3 of a minimum of 12, 8 and 12 foreseen visits, respectively. Attrition was most pronounced on visits at 35-40 weeks of gestation.

**Figure 3:**
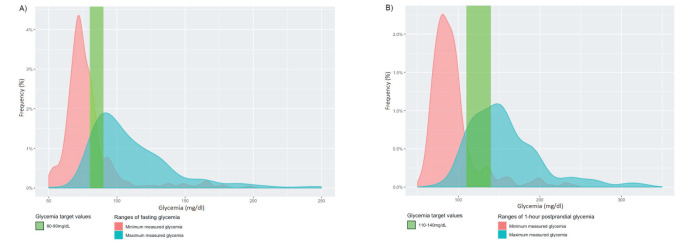
Glycemia measured at home. **Panel A.** Density curves of minimum and maximum fasting blood glycemia. **Panel B.** Density curves minimum and maximum 1-hour postprandial blood glycemia.

Among patients with DM, 39 (58.2%) did not receive pharmacotherapy, 21(31.3%) were on metformin only, 6 (9%) were on metformin and insulin, and 1 (1.5%) was on insulin only (**Table 2**). Metformin was given at an average dose <500 mg/d (**Table 2**). During follow-up, medians (IQR) for minimum and maximum fasting glycemia were 74 (68-81) and 102 (89-125) mg/dL (Figure 3A), and medians (IQR) for minimum and maximum 1-hour postprandial glycemia were 86 (76-99) and 150 (124-182) mg/dL, indicating an overall reasonable control for women who attended follow-up visits (**Figure 3**, Panel B). Most patients with HDP required pharmacologic monotherapy for BP (**Table 2**). Mean systolic BP during follow-up ranged from 115.1 ± 16.9 to 120.8 ± 16.5 mm Hg and mean BP pressure ranged from 77.2 ± 12.5 to 79.0 ± 12.9 mm Hg (**Table 2**).

### Perinatal outcomes

A total of 422 (59%) women were delivered at the hospital: 282 (66.8%) controls, 37 (8.8%) with DM, 78 (18.5%) with HDP, and 25 (5.9%) with DM/HDP (**Figure 1**, Panel B). The mean gestational age was highest among controls and lowest among women with HDP (Table 3). Preterm birth <32 weeks was more common among women the HDP group than among the control, DM, and DM/HDP groups (**Table 3**). Vaginal deliveries occurred more commonly in controls than in women with DM and/or HDP (**Table 3**).

**Table 3:** Obstetrical, maternal and neonatal outcomes

Total (N = 422)	Controls (N = 282)	DM (N = 37)	HDP (N = 78)	DM/HDP (N = 25)	*P-*value*
Gestational age at delivery, weeks, mean ± SD (n = 168)	39 ± 2.4	39.4 ± 3.0	37.9 ± 3.9	38.7 ± 2.1	0.893
<32weeks, n (%)	1 (0.4)	0	4 (5.1)	0	0.023
32-36 weeks, n (%)	14 (5.0)	3 (8.1)	6 (7.7)	1 (4.0)	0.529
≥37 weeks, n (%)	99 (35.1)	10 (27.0)	23 (29.5)	7 (28.0)	0.108
Delivery mode:					
Vaginal, n (%)	227 (80.4)	21 (56.8)	60 (76.9)	16 (64.0)	<0.001
Cesarean delivery, n (%)	19 (6.7)	10 (27.0)	15 (19.2)	5 (20.0)	<0.001
Forceps, n (%)	1 (0.4)	0	0	0	1
Maternal blood glycemia, mean ± SD (n = 3)	n/a	72	n/a	72 ± 5	0.948
Maternal blood pressure (n = 337):					
BP≥160/110 mm Hg, n (%)	n/a	n/a	20 (25.6)	5 (20.0)	0.789
Systolic BP, mmHg, mean ± SD	116 ± 12	120 ± 11	144 ± 19	143 ± 21	<0.001
Diastolic BP, mmHg, mean ± SD	70 ± 10	74 ± 10	91 ± 17	89 ± 13	<0.001
Preeclampsia, n (%)	0	0	45 (57.7)	13 (52.0)	<0.001
Eclampsia, n (%)	0	0	5 (6.4)	0	0.002
Small for gestational age, n (%)	11 (3.9)	0	3 (3.8)	2 (8.0)	0.320
Large for gestational age, n (%)	21 (7.4)	6 (16.2)	6 (7.7)	1 (4.0)	0.170
Birth weight (n = 362):					
<2500 g, n (%)	20 (7.1)	1 (2.7)	20 (25.6)	3 (12.0)	<0.001
2,500-4000 g, n (%)	212 (75.2)	24 (64.9)	53 (67.9)	17 (68.0)	0.004
>4000 g, n (%)	6 (2.1)	5 (13.1)	1 (1.3)	0	0.008
Obstetrical complications (n = 366)					
Shoulder dystocia, n (%)	0	0	1 (1.3)	0	0.339
Perinatal asphyxia, n (%)	0	0	0	0	1
Neonatal hypoglycemia, n (%) (n = 64)	n/a	1 (2.7)	0	2 (8.0)	0.366
Intravenous dextrose, n (%)	–	1 (100)	–	1 (100)	1
Neonatal complications (n = 363):					
Fetal death, n (%)	2 (0.7)	1 (2.7)	1 (1.3)	2 (8.0)	0.017
Neonatal death, n (%)	3 (1.1)	0	3 (3.8)	3 (12.0)	0.013

BP – blood pressure, DM – diabetes mellitus, HDP – hypertensive disorders of pregnancy, SD – standard deviation

*P-value for difference between groups.

While 39 women had an HDP at the time of first visit, 30 women developed HDP during follow-up (n = 69 women at the end of antepartum follow-up), and 51 manifested with HDP at time of delivery (n = 120 by time of delivery, **Table 2**). Among women with HDP, systolic BP was above target upon presentation for delivery, with at least 20% presenting with severe hypertension (**Table 3**). Preeclampsia affected 58 (13.7%) women (n = 45 in the HDP group, n = 15 in the DM/HDP group), which represented 56.3% of all HDP deliveries (Table 3). Five women in the HDP group had eclampsia and for three of them, eclamptic seizures were the first manifestation of HDP (**Table 3**).

Babies with low birth weight (<2500 g) were more common in the HDP than in the control and DM groups (**Table 3**). However, there was no difference in the number of babies born SGA (**Table 3**). Macrosomia was more frequent among women with DM than in the control and HDP groups (**Table 3**). Primarily due to a shortage of pediatric staff, protocol deviation for hypoglycemia screening in infants of diabetic mothers occurred in 19 such that only 43 of 62 neonates who would have had a blood glucose check at 1 hour of life were actually tested. Neonatal hypoglycemia was reported in three (7%) neonates of 43 mothers with DM and DM/HDP, two of which required intravenous dextrose (**Table 3**). Perinatal mortality. designating fetal and neonatal deaths, affected 15 (36 per 1000) deliveries, including 5 (18 per 1000) deliveries in the control, 1 (27 per 1000) delivery in the DM, 4 (51 per 1000 deliveries) deliveries in the HDP, and 5 (200 per 1000) deliveries in the DM/HDP groups. Missing information for variables included in **Table 3** can be found in Table S2 of the **Online Supplementary Document**.

## DISCUSSION

We conducted a prospective cohort study of pregnant women with and without DM and HDP in in a regional hospital in Haiti. We reported on the local prevalence of these NCDs and their associated perinatal outcomes. We found that DM and HDP affected 11.3% and 16.8% of women in our cohort, respectively. While BP management was optimized during antepartum follow-up, systolic BP was above target upon presentation for delivery among women with HDP. Infants exposed to maternal DM had more macrosomia than those in the control, HDP, and DM/HDP groups. More early preterm births <32 weeks occurred among women with HDP, resulting in a higher proportion of infants with low birth weight in this group. The overall perinatal mortality rate in our cohort was mostly driven by deaths among infants of mothers with DM and/or HDP. This study will help to prepare future refinements to optimize the management of NCDs in pregnancy in order to reduce adverse maternal and neonatal outcomes.

### Diabetes in pregnancy

In our cohort, obesity was associated with HDP, yet not with gestational DM. Risk factors for hyperglycemia may vary in a context of food insecurity and undernutrition [22], which further supports universal screening for gestational DM in LMIC, as traditional cardiometabolic risk factors may not apply. High rates of macrosomia and perinatal mortality among women with DM in our study suggests that antenatal hyperglycemia likely persisted for some women. High rates of macrosomia and perinatal mortality among women with DM in LMIC have also been reported by others [23-25]. While the median maximum self-measured glycemia of women who attended follow-up was 10 mg/dL above target, the median maximum glycemia of women who were not present at follow-up may have been higher, leading to an underestimation of suboptimal glycemic control in the cohort. Although we found that less neonates had macrosomia among women with DM/HDP than DM alone, a normal weight at birth may not have been synonymous with metabolic health. Indeed, the effects of hyperglycemia on fetal weight may have been offset by the presence of intrauterine growth restriction.

### Hypertensive disorders of pregnancy

In Latin America and the Caribbean HDP account for 22.2% of all maternal deaths [26]. The incidence of preeclampsia among women delivered in hospital in our study was comparable to the incidence reported by others [27], which was almost three times higher than the estimated global incidence of ~ 5% [28]. This proportion should however be interpreted with caution since 41% of women in our cohort were not delivered in hospital. The high rates of preeclampsia in our study may warrant consideration for preventive measures such as universal prophylaxis with low-dose aspirin [29].

Between the last scheduled antenatal visit and delivery, 40 women from the control group developed HDP, preeclampsia being the most common diagnosis. Multifactorial delays in triage, transport, and treatment of women with HDP may explain the increased burden of adverse outcomes associated with HDP in LMIC [30]. Since most women in our cohort were diagnosed with preeclampsia at time of admission for delivery, a timelier diagnosis of preeclampsia could possibly improve maternal and neonatal outcomes. In addition, we observed a high incidence of eclampsia in our study, which may be decreased with tighter BP management at time of admission for delivery, and a low threshold to initiate magnesium sulfate prophylaxis. Community level interventions with facility enhancement have been suggested to decrease preeclampsia-related adverse outcomes [30,31].

### Challenges in the implementation of care delivery algorithms

Our study illustrates some challenges in caring for NCDs in low-income countries [2]. To overcome limitations in laboratory equipment, we used portable glucometer for oGTT and point-of-care urinary dipstick tests for proteinuria assessment. The evaluation of women with preeclampsia was limited by unavailability of kidney and liver function tests, despite both being featured in the Second WHO Model List of Essential In Vitro Diagnostics [32]. We had to contend with supply shortages, as the provision of Glucola, portable glucometers, and strips, was intermittently interrupted during the study period. Shortages in insulin occurred despite it being on the WHO Model List of Essential Medicine [33]. We obtained additional insulin from neighbouring pharmacy and diabetes clinic when the insulin was unavailable. Human shortages were present at multiple steps of patient care. Intrapartum maternal and neonatal glucose monitoring protocols were particularly challenging. Such barriers may represent important hurdles to overcome in order to insure optimal NCD care in LMIC.

### Strengths and limitations

Our study had several strengths. We introduced an innovative clinical care delivery program for DM in pregnancy using home glucose monitoring, oral agents, and insulin in a public hospital in Haiti where no infrastructure had previously been in place. Outcomes reported reflected a standardized approach to the management of NCDs in pregnancy. We provided valuable insights on areas to prioritize for the development and implementation of care delivery models for NCDs in pregnancy in LMIC. Our study had some limitations. Only 59% of women delivered in hospital and those who delivered outside of our health care facility may have experienced different outcomes. Moreover, neonatal glucose levels were not measured in all infants and the true prevalence of neonatal hypoglycemia was likely underestimated. We could not measure the impact of our intervention on maternal and neonatal outcomes since data prior to the DYAMAN study initiation was not available. We may have been underpowered to detect differences between groups for rare outcomes, such as neonatal hypoglycaemia. Our study population was a sample of pregnant women attending the antenatal clinic of a regional hospital in Saint-Marc and results may not be generalizable to the rest of the country’s pregnant population.

In conclusion, we showed that NCDs in pregnancy in Haiti were prevalent, challenging to care for, and important drivers of adverse maternal and perinatal outcomes. Further research is needed to assess local barriers, including economic barriers, to follow-up and treatment adherence during antepartum management of NCDs. Moreover, barriers to treatment escalation for maternal hyperglycemia, including physicians’ and patients’ experiences and perspectives must be explored. Identifying methods to increase health care contact after 36 weeks of gestation may partially prevent maternal and perinatal adverse outcomes resulting from late presentation to care. Finally, for gestational DM care delivery programs to be sustainable, the development of local capacity to supply diagnostic testing must be prioritized. The WHO established NCDs as a priority in the Global Health Action Plan through their “Strategy for Prevention and Control of NCD’s 2013-2020” [34]. Despite a global recognition of this pandemic, NCDs in low-income countries being the recipients of only 1.3% of development assistance for health in 2015 remain grossly underfunded [35]. We call for greater funding of research initiatives and health care delivery programs aimed at combatting health inequities disproportionately affecting women and children globally.

## Additional material

Online Supplementary Document

## References

[1] World Health Organization. Global status report on noncommunicable diseases 2010: WHO. Available: https://www.who.int/nmh/publications/ncd_report2010/en/#.XR-t1h3QcD4.mendeley. Accessed: 5 July 2019.

[2] Allotey P, Davey T, Reidpath DD. NCDs in low and middle-income countries - assessing the capacity of health systems to respond to population needs. BMC Public Health. 2014;14 Suppl 2(Suppl 2):S1. 10.1186/1471-2458-14-S2-S1 PMC4120152 25082328

[3] Metzger BE Lowe LP Dyer AR Trimble ER Chaovarindr U Coustan DR Hyperglycemia and adverse pregnancy outcomes. N Engl J Med. 2008;358:1991-2002. 10.1056/NEJMoa0707943 18463375

[4] Brown MA Lindheimer MD de Swiet M Van Assche A Moutquin JM The classification and diagnosis of the hypertensive disorders of pregnancy: statement from the International Society for the Study of Hypertension in Pregnancy (ISSHP). Hypertens Pregnancy. 2001;20:IX-XIV. 10.3109/10641950109152635 12044323

[5] Feig DS Berger H Donovan L Godbout A Kader T Keely E Diabetes and Pregnancy. Can J Diabetes. 2018;42 Suppl 1:S255-S282. 10.1016/j.jcjd.2017.10.038 29650105

[6] Ray JG Vermeulen MJ Schull MJ Redelmeier DA Cardiovascular health after maternal placental syndromes (CHAMPS): population-based retrospective cohort study. Lancet. 2005;366:1797-803. 10.1016/S0140-6736(05)67726-4 16298217

[7] Grandi SM Filion KB Yoon S Ayele HT Doyle CM Hutcheon JA Cardiovascular Disease-Related Morbidity and Mortality in Women With a History of Pregnancy Complications. Circulation. 2019;139:1069-79. 10.1161/CIRCULATIONAHA.118.036748 30779636

[8] World Health Organization. A prioritized research agenda for prevention and control of NCDs: CVD, cancer, chronic respiratory disease, diabetes 2015. Available: https://www.who.int/cardiovascular_diseases/publications/ncd_agenda2011/en/#.XR-5YzgROOk.mendeley. Accessed: 5 July 2019.

[9] WHO, UNFPA, World Bank Group, and United Nations Population Division, Group MMEI-A. Maternal mortality in 1990-2015, Haiti. Available: http://www.who.int/gho/maternal_health/countries/hti.pdf. Accessed: 1 February 2018.

[10] The World Bank 2017. Available: https://data.worldbank.org/indicator/sh.dyn.nmrt?most_recent_value_desc=false. Accessed: 11 July 2019.

[11] Cayemittes M, Busangu MF, Bizimana JD, Barrère B, Sévère B, Cayemittes V, et al. Enquête Mortalité, Morbidité et Utilisation des Services EMMUS-V 2012. Available: https://mspp.gouv.ht/site/downloads/EMMUS%20V%20document%20final.pdf

[12] Word Health Organization. Diagnostic criteria and classification of hyperglycaemia first detected in pregnancy.2013. Available: www.who.int/diabetes/publications/Hyperglycaemia_In_Pregnancy/en/#.XR-78-RH9KI.mendeley. Accessed: 5 July 2019. 24199271

[13] Pardo S Dunne N Simmons DA Using Radar Plots to Demonstrate the Accuracy and Precision of 6 Blood Glucose Monitoring Systems. J Diabetes Sci Technol. 2017;11:966-9. 10.1177/1932296817713026 28604065 PMC5950999

[14] Klaff LJ Brazg R Hughes K Tideman AM Schachner HC Stenger P Accuracy evaluation of contour next compared with five blood glucose monitoring systems across a wide range of blood glucose concentrations occurring in a clinical research setting. Diabetes Technol Ther. 2015;17:8-15. 10.1089/dia.2014.0069 25260047

[15] Hod M Kapur A Sacks DA Hadar E Agarwal M Di Renzo GC The International Federation of Gynecology and Obstetrics (FIGO) Initiative on gestational diabetes mellitus: A pragmatic guide for diagnosis, management, and care. Int J Gynaecol Obstet. 2015;131 Suppl 3:S173-211. 10.1016/S0020-7292(15)30033-3 26433807

[16] Balaji V Madhuri BS Paneerselvam A Arthi T Seshiah V Comparison of venous plasma glucose and capillary whole blood glucose in the diagnosis of gestational diabetes mellitus: a community-based study. Diabetes Technol Ther. 2012;14:131-4. 10.1089/dia.2011.0060 21992269

[17] de Greeff A Beg Z Gangji Z Dorney E Shennan AH Accuracy of inflationary versus deflationary oscillometry in pregnancy and preeclampsia: OMRON-MIT versus OMRON-M7. Blood Press Monit. 2009;14:37-40. 10.1097/MBP.0b013e32831e305d 19252437

[18] Magee LA Pels A Helewa M Rey E von Dadelszen P Diagnosis, evaluation, and management of the hypertensive disorders of pregnancy. Pregnancy Hypertens. 2014;4:105-45. 10.1016/j.preghy.2014.01.003 26104418

[19] National Collaborating Centre for Women’s. Children's Health. National Institute for Health and Care Excellence: Clinical Guidelines. Diabetes in Pregnancy: Management of Diabetes and Its Complications from Preconception to the Postnatal Period. London: National Institute for Health and Care Excellence (UK); 2015. 25950069

[20] Harris PA Taylor R Thielke R Payne J Gonzalez N Conde JG Research electronic data capture (REDCap)–a metadata-driven methodology and workflow process for providing translational research informatics support. J Biomed Inform. 2009;42:377-81. 10.1016/j.jbi.2008.08.010 18929686 PMC2700030

[21] Harris PA Taylor R Minor BL Elliott V Fernandez M O’Neal L The REDCap consortium Building an international community of software platform partners. J Biomed Inform. 2019;95:103208. 10.1016/j.jbi.2019.103208 31078660 PMC7254481

[22] Miranda JJ Barrientos-Gutierrez T Corvalan C Hyder AA Lazo-Porras M Oni T Understanding the rise of cardiometabolic diseases in low- and middle-income countries. Nat Med. 2019;25:1667-79. 10.1038/s41591-019-0644-7 31700182

[23] John CO Alegbeleye JO Otoide AO Foeto-maternal outcome of diabetes in a tertiary health facility in Nigeria. African J Diabetes Med. 2015;23:13-6.

[24] Utz B Assarag B Smekens T Ennassiri H Lekhal T El Ansari N Detection and initial management of gestational diabetes through primary health care services in Morocco: An effectiveness-implementation trial. PLoS One. 2018;13:e0209322. 10.1371/journal.pone.0209322 30592751 PMC6310282

[25] Thomas N Chinta AJ Sridhar S Kumar M Kuruvilla KA Jana AK Perinatal outcome of infants born to diabetic mothers in a developing country–comparison of insulin and oral hypoglycemic agents. Indian Pediatr. 2013;50:289-93. 10.1007/s13312-013-0096-y 23255686

[26] Say L Chou D Gemmill A Tuncalp O Moller AB Daniels J Global causes of maternal death: a WHO systematic analysis. Lancet Glob Health. 2014;2:e323-33. 10.1016/S2214-109X(14)70227-X 25103301

[27] Raghuraman N March MI Hacker MR Modest AM Wenger J Narcisse R Adverse maternal and fetal outcomes and deaths related to preeclampsia and eclampsia in Haiti. Pregnancy Hypertens. 2014;4:279-86. 10.1016/j.preghy.2014.09.002 26104817

[28] Abalos E Cuesta C Grosso AL Chou D Say L Global and regional estimates of preeclampsia and eclampsia: a systematic review. Eur J Obstet Gynecol Reprod Biol. 2013;170:1-7. 10.1016/j.ejogrb.2013.05.005 23746796

[29] Ayala NK Rouse DJ A Nudge Toward Universal Aspirin for Preeclampsia Prevention. Obstet Gynecol. 2019;133:725-8. 10.1097/AOG.0000000000003167 30870274

[30] von Dadelszen P Ansermino JM Dumont G Hofmeyr GJ Magee LA Mathai M Improving maternal and perinatal outcomes in the hypertensive disorders of pregnancy: a vision of a community-focused approach. Int J Obstet Gynecol. 2012;119 Suppl 1:S30-4. 10.1016/j.ijgo.2012.03.012 22884823

[31] von Dadelszen P Bhutta ZA Sharma S Bone J Singer J Wong H The Community-Level Interventions for Pre-eclampsia (CLIP) cluster randomised trials in Mozambique, Pakistan, and India: an individual participant-level meta-analysis. Lancet. 2020;396:553-63. 10.1016/S0140-6736(20)31128-4 32828187 PMC7445426

[32] World Health Organization. Second WHO Model List of Essential In Vitro Diagnostics. Geneva: World Health Organization; 2019.

[33] World Health Organization. WHO Model List of Essential Medicines: The World Health Organization; 2017. Available: https://apps.who.int/iris/bitstream/handle/10665/273826/EML-20-eng.pdf?ua=1. Accessed: 11 July 2019.

[34] Zouein FA Altara R Chen Q Lesnefsky EJ Kurdi M Booz GW Pivotal Importance of STAT3 in Protecting the Heart from Acute and Chronic Stress: New Advancement and Unresolved Issues. Front Cardiovasc Med. 2015;2:36. 10.3389/fcvm.2015.00036 26664907 PMC4671345

[35] Kolodziej AR Rajagopala N Guglin M Retrobulbar Hematoma After Heart Transplantation: Case Report and Literature Review. Transplant Proc. 2015;47:2788-90. 10.1016/j.transproceed.2015.09.034 26680096

