# Supplementary material for: Prevalence and perinatal outcomes of non-communicable diseases in pregnancy in a regional hospital in Haiti: A prospective cohort study
Source: J Glob Health. 2021 Apr 17;11:04020. doi: 10.7189/jogh.11.04020 (PMC8053393; doi:10.7189/jogh.11.04020)
Supplement: Online Supplementary Document [file jogh-11-04020-s001.pdf]

Supplementary Appendix S1: Visit Frequency based on diagnosis

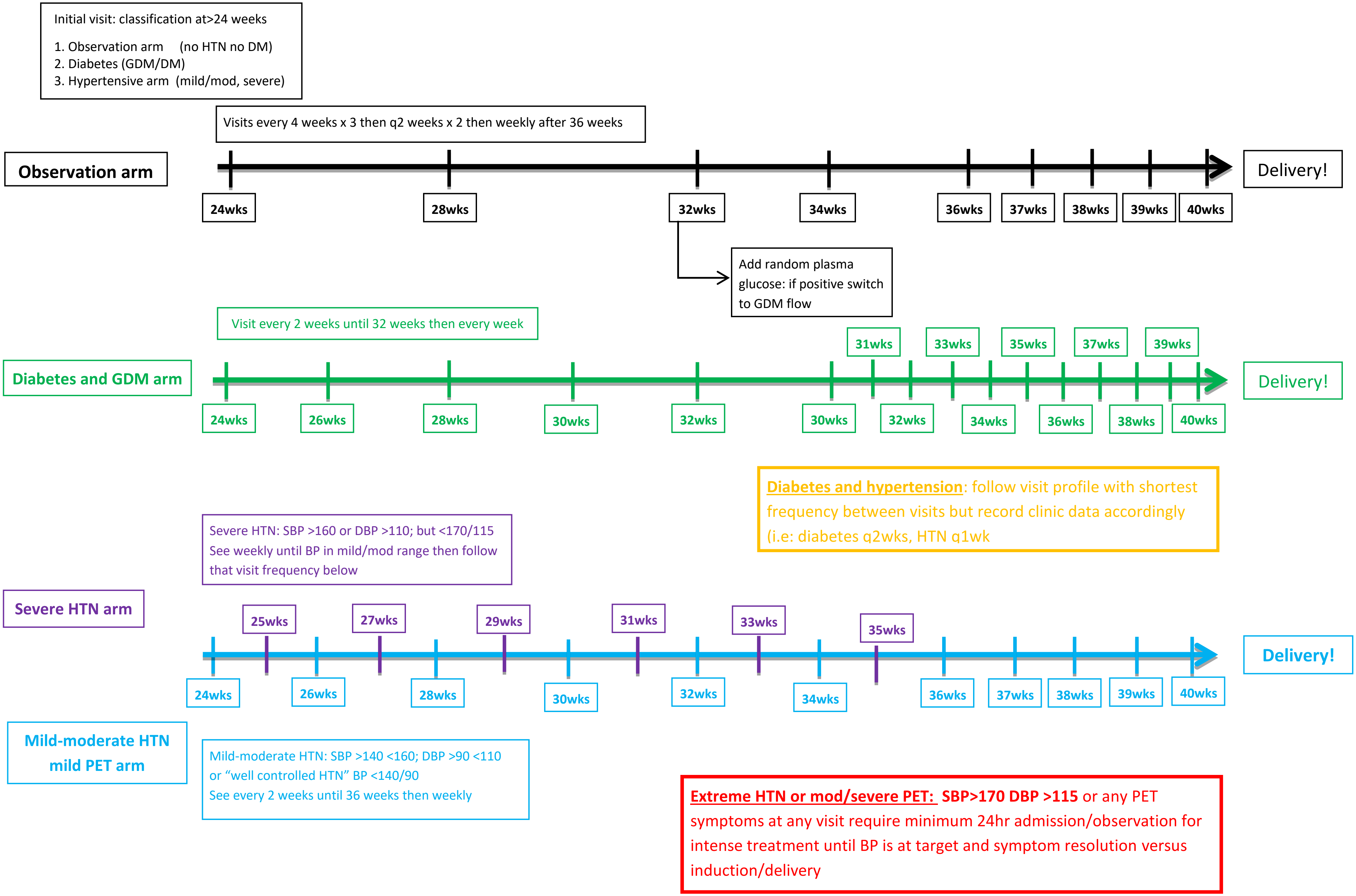

Supplementary Appendix D2: Diabetes Mellitus diagnosis, classification and treatment protocols

Diagnosis and Classification of Diabetes Mellitus type at 1st antenatal visit (24 to 28 weeks)

1st antenatal visit				
**24-28 Weeks**	75g oral Glucose Tolerance Test (oGTT) (fasting, 1hr + 2hr post)	Pre-existing DM	Fasting BG > 126 mg/dL **or** 2hr oGTT: >200 mg/dL	→ Start protocol “insulin”
		Gestational DM	BG >92 mg/dL and < 126 mg/dL<br>1hr post oGTT >180 mg/dL<br>2hr post pGTT > 153 mg/dL and < 200 mg/dL	→ Start protocol “Gestational DM”
		Normal	All values below the above thresholds	Routine care

→ If risk markers: macrosomia, poly hydramnios – repeat oGTT

75g oGTT - Algorithm :

- Ensure that patient has been fating for > 8 hrs (1 night). Document hour of last meal
- Advise patient that she cannot eat, drink, or smoke while the test is ongoing
- If patient not fasting, she can come back for testing on the following day or week

Step #1: Perform capilary glycemia, which will be the ‘fasting blood glucose’

↓

Step#2 2: Administrer 75g glucose (Glucola), which patient has to drink within 5 mins

↓

Step #3: Perform capillary blood glucose at 1 and 2hr following glucose load, document les results and follow the above algorithm

TREATMENT PROTOCOL WITHOUT INSULIN

Pre-existing or gestational DM new diagnosis

Nutrition, diet, exercice and

Teaching on home BG monitoring

Antenatal visit every 1-2 weeks, fasting

Review diet, exercise, adherence to medications

Fasting BG

Fasting or AC BG < 90 mg/dL

PC 1hr <140 mg/dL

Fasting or AC BG > 90 mg/dL and <126 mg/dL

PC 1hr > 140mg/dL

AT TARGET

Continue monitoring BG daily at home

NOT AT TARGET

Start metformin 500 mg PO BID x 7 days, and increase to 1g PO BID

If already on metformin 1g PO BID start insulin protocol and add insulin to metformin

If fasting or AC BG**> 126mg/dL**
Start metformin **and** insulin

Start insulin protocol and + continue metformin

Start treatment if >2 BG not at target

TREATMENT PROTOCOL WITH INSULIN

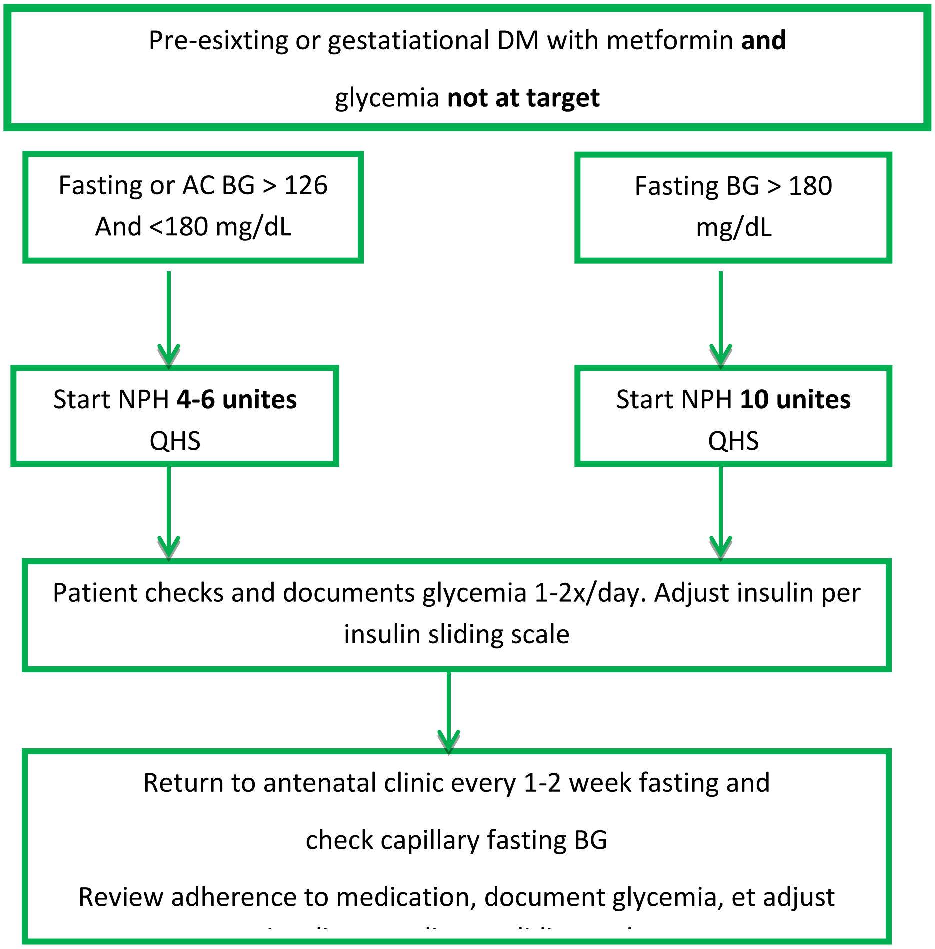

INSULIN SLIDING SCALE

HUMULIN R BEFORE MEALS AND NPH AT BEDTIME

If Glycemia prior to breakfast:

>140	Add **6** units to the NPH dose taken at bedtime
116-140	Add **4**units to the NPH dose taken at bedtime
100-115	Add **2** units to the NPH dose taken at bedtime
91-100	Add **1** units to the NPH dose taken at bedtime
**80-90**	**Prenez la même dose de NPH prise la nuit précédente**
<80	Or nighttime hypoglycemia—**reduce** the NPH dose taken on the night before by **2 units**

Si la glycémie 1 heure après le petit-déjeuner est :

>170	Add **2** units to the dose of Humulin R before breakfast
141-170	Add **1** units to the dose of Humulin R before breakfast
**110-140**	**Take the same dose of Humulin R as taken at breakfast on the preceding day**
<110	Or morning hypoglycemia—**reduce** the Humulin R dose taken at breakfast by **2 units**

Si la glycémie 1 heure après le déjeuner est :

>170	Add **2** units to the dose of Humulin R before lunch
141-170	Add **1** unit to the dose of Humulin R before lunch
**110-140**	**Take the same dose of Humulin R as taken at lunch on the preceding day**
<110	Or afternoon hypoglycemia—**reduce** the Humulin R dose taken at lunch by **2 units**

Si la glycémie 1 heure après le dîner est :

>170	Add **2** units to the dose of Humulin R before diner
141-170	Add **1** to the dose of Humulin R before diner
**110-140**	**Take the same dose of Humulin R as taken at diner on the preceding day**
<110	Or evening hypoglycemia—**reduce** the Humulin R dose taken at diner by **2 units**

****If elevated BG because of excess snack or meal with too much carbohydrates, correct the provoking factor and keep the same insulin dose.**

Target bloog glucose:

**Fasting:**	**80-90 mg/dL**
**1h after meal**	**110-140 mg/dL**

Supplementary Appendix S3: Intrapartum Diabetes Management Protocol

Daily quantity of Insulin

≥30 units

Diet: clear liquids
IV : D5W at 75-100cc/hr
Glycaemias : q 2 hrs until delivery
Insulin: SC with sliding scale

Start INSULIN :
Calculate initial insulin dose: Daily insulin quantity divided by 2 and again by 24 to reach initial hourly dose.
EX : Women ataking 50 units am and 50 units pm → total daily dose of 100 units /24h → 100÷2 = 50 → 50÷24 = 2.08 → **~2 units q 2 hr**

< 30 units

Diet: regular
IV : as needed
Glycaemias : q 2 hrs x 2

Glycemia ≥ **126**

Glycaemia **70-125**

Glycaemia mg/dL	Insuline units SQ (pte >30 U/jr)	Insuline units SQ (pt <30 U/jr)	Liquides IV etc
<70	Bolus D50, stop insuline		D5W @ 75-100cc/hr
70-126	Continue with same dose	0 unit	
127-140	increase the dose by **1** unit	**1** unit	
141-160	increase the dose by **2** units	**2** units	
161-180	increase the dose by **3** units	**3** units	
181-200	increase the dose by **4** untiés	**4** units	
≥200	Glycaemias q1H, cesser soluté, débuter insuline IV (voir ordonnances)		

Glycaemias q 4 hrs until delivery with target as above

DELIVERY

Stop insulin sliding scae ; Diet : regular if tolerated ;
IV: D5W at 100-125cc/h x 2 hrs; Glycaemia après 2 hrs

Glycaemia **<70** ou **>180** call MD to treat and follow glycaemia q 2 hrs

Glycaemia **70-180** and patient tolerating po→ stop IV and repeat glycaemia in 2 hrs

Glycaemia **70-180** :
Stop checking glycaemia today

DELIVERY

Diet: regular, IV fluids if not eating; glycaemia in 2 hrs

Glycaemia **<70** ou **>140** call MD to treat glycaemia

Glycaemia **70-140**: Stop checking glycaemias today

Fasting glycaemia on following morning

Supplementary Appendix S4: Neonatal Hypoglycemia Protocol

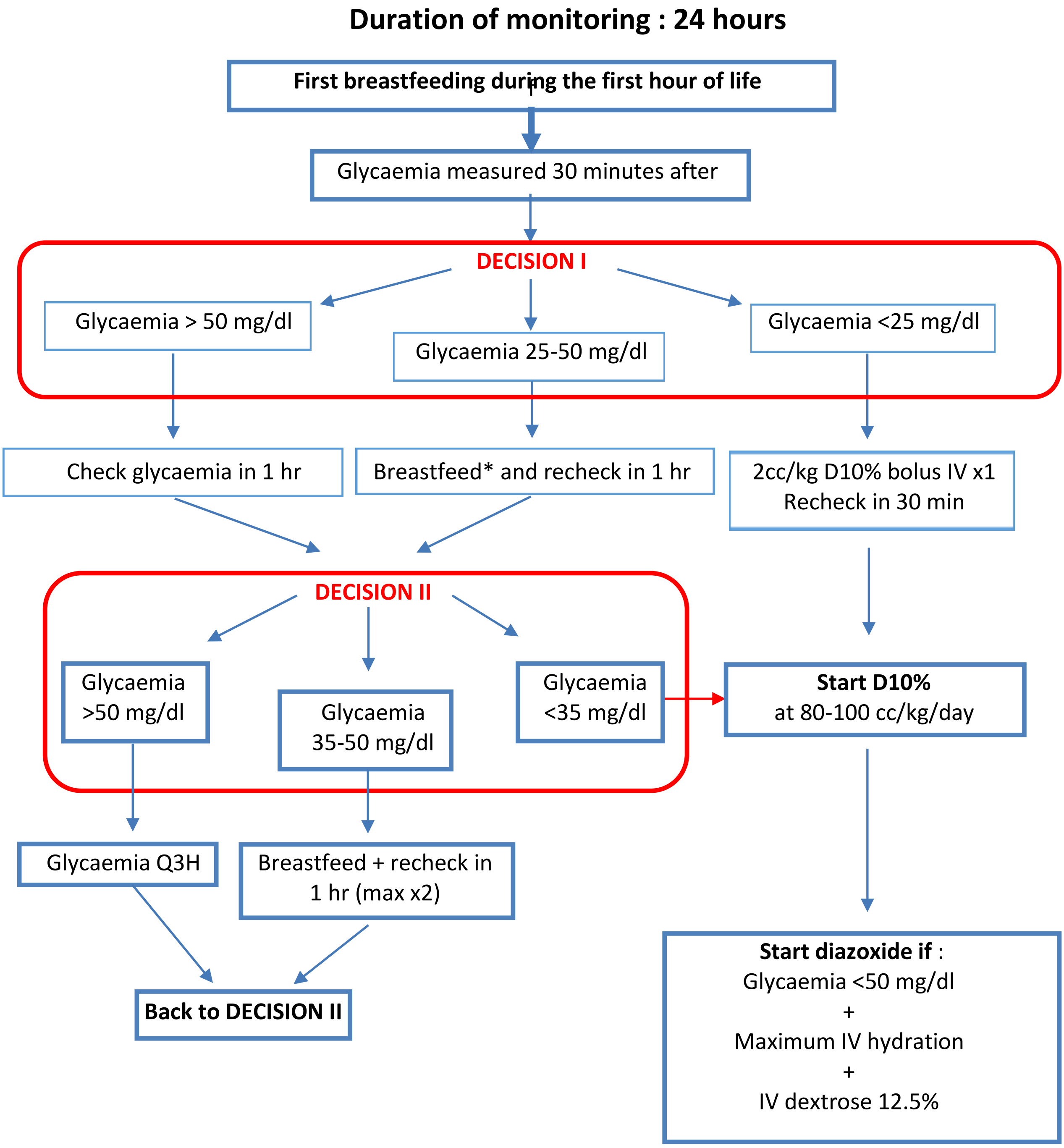

*Clinical judgement to decide whether maternal or formula milk indicated

Supplementary Table S1. Missing values in baseline characteristics across group categories

Variable	Control N=544 n (%)	DM N=51 n (%)	HDP N=90 n (%)	DM/HDP N=30 n (%)
Gestational age at initial visit	4 (0.7)	0	1 (1.1)	0
Age	7 (1.3)	0	2 (2.2)	0
BMI	10 (1.8)	2 (3.9)	1 (1.1)	0
Prior pregnancies	2 (0.4)	0	0	1(3.3)
Live children	1 (0.3)	0	1 (1.7)	0
Matrimonial status	1 (0.2)	0	0	0
Education length	0	1 (2.0)	0	0
House built with concrete, n (%)	3 (0.6)	0	0	0
Food insecurity, n (%)	5 (0.9)	0	0	0
Current smoker, n (%)	3 (0.6)	0	0	0
Current alcohol use, n (%)	3 (0.6)	0	0	0
Comorbidities				
One elevated blood pressure in lifetime, n (%)	3 (0.6)	0	1 (1.7)	0
Preexisting diabetes, n (%)	3 (0.5)	0	0	0
Obstetrical history				
Gestational hypertension, n (%)	3 (1.0)	0	0	0
Preeclampsia, n (%)	3 (1.0)	0	0	0
Prior testing for gestational diabetes, n (%)	8 (2.5)	0	0	0

BMI= Body mass index, DM = Diabetes Mellitus, HDP = Hypertensive disorder of pregnancy, SD= Standard deviation

Supplementary Table S2. Missing values in maternal and neonatal outcomes results

Total (N=422)	Controls N=282 Missing n (%)	DM N=37 Missing n (%)	HDP N=79 Missing n (%)	DM/HDP N=25 Missing n (%)
Gestational age at delivery	168 (59.6)	24 (64.9)	45 (57.7)	17(68.0)
Maternal blood glycemia	--	36 (97.3)	--	23 (92.0)
Maternal blood pressure				
Systolic BP	61 (21.6)	12 (32.4)	8 (10.2)	4 (16.0)
Diastolic BP	61 (21.6)	12 (32.4)	8 (10.2)	4 (16.0)
Preeclampsia, n (%)	49 (17.4)	9 (24.3)	5 (6.4)	4 (16.0)
Eclampsia, n (%)	49 (17.4)	9 (24.3)	6 (7.7)	4 (16.0)
Delivery mode	35 (12.4)	6 (16.2)	3 (3.8)	4 (16.0)
Small for gestational age, n (%)	174 (61.7)	24 (64.9)	46 (58.9)	17 (68.0)
Large for gestational age, n (%)	174 (61.7)	24 (64.9)	46 (58.9)	17 (68.0)
Birth weight (n=362)	44 (15.6)	7 (18.9)	4 (5.1)	5 (20.0)
Obstetrical complications	40 (14.1)	7 (18.9)	5 (6.4)	4 (16.0)
Neonatal hypoglycemia, n (%)	n/a	12 (32.4)	55 (70.5)	7 (28.0)
Intravenous dextrose, n (%)	-	0	-	1 (50)
Neonatal complications	45 (16.0)	6 (16.2)	3 (3.8)	3 (12.0)

BP= Blood pressure, DM= Diabetes mellitus, HDP= Hypertensive disorders of pregnancy, SD= Standard deviation
